# Modification of Body-Related Attentional Bias through Virtual Reality and Eye-Tracking in Healthy Participants: Implications for Anorexia Nervosa Treatments

**DOI:** 10.3390/brainsci13050764

**Published:** 2023-05-05

**Authors:** Helena Miquel-Nabau, Natalia Briseño-Oloriz, Bruno Porras-Garcia, Mariarca Ascione, Franck-Alexandre Meschberger-Annweiler, Marta Ferrer-Garcia, Manuel Moreno-Sanchez, Eduardo Serrano-Troncoso, Marta Carulla-Roig, José Gutiérrez Maldonado

**Affiliations:** 1 Department of Clinical Psychology and Psychobiology, Institute of Neurosciences, University of Barcelona, 08035 Barcelona, Spain; helena.mn29@gmail.com (H.M.-N.); natalia.brisenoloriz@gmail.com (N.B.-O.); ascione.m@ub.edu (M.A.); franck.meschberger@ub.edu (F.-A.M.-A.); martaferrerg@ub.edu (M.F.-G.); 2 Department of Population Health Science, University of Utah School of Medicine, 295 Chipeta Way, Salt Lake City, UT 84112, USA; brnopg91@gmail.com; 3 Department of Cognition, Development and Educational Psychology, University of Barcelona, Passeig de la Vall d’Hebron 171, 08035 Barcelona, Spain; m_moreno@ub.edu; 4 Child and Adolescent Psychiatry and Psychology Department, Hospital Sant Joan de Déu of Barcelona, 08950 Esplugues de Llobregat, Spain; eduardo.serrano@sjd.es (E.S.-T.); marta.carulla@sjd.es (M.C.-R.)

**Keywords:** attentional bias, anorexia nervosa, virtual reality, body image, eye-tracking

## Abstract

Cognitive biases have a significant impact on the etiology and treatment of eating disorders (EDs). These biases, including selective attentional bias (AB) to disliked body parts, may reinforce concerns about body shape, fear of gaining weight and body image disturbances and may contribute to dietary restriction and restraint. Decreasing AB could reduce core symptoms in anorexia nervosa (AN). This study represents a preliminary exploration aiming to assess whether AB towards weight-related (WR) and non-weight-related (NW) body parts could be reduced through an AB modification task in a virtual reality (VR) environment in healthy participants. A total of 54 female participants, aged 22.98 ± 1.89, were recruited. The task consisted of directing the participants’ attention towards all body parts equally in a VR setting. Eye-tracking (ET) measurements (complete fixation time [CFT] and number of fixations [NF]) were made before and after the task. The results showed a significant reduction of the AB in the two groups with an initial AB towards WR body parts or towards NW body parts. Participants showed a tendency to more balanced (non-biased) attention after the intervention. This study provides evidence of the usefulness of AB modification tasks in a non-clinical sample.

## 1. Introduction

Anorexia nervosa (AN) is a complex disorder with many factors involved in its development and maintenance. Adolescent girls and young adult women are particularly at risk. Intense fear of gaining weight, persistent insufficient-intake restrictions, excessive weight loss, body-image disturbances and a dysfunctional body self-perception (BIDs) are all common characteristics of AN [1]. Individuals with anorexia nervosa (AN) often exhibit significant disturbances in their cognitive and emotional functioning. They also frequently experience serious medical and psychiatric comorbidities [2]. The disorder tends to have a relapsing or protracted course in adults and older adolescents, and if left untreated, high levels of disability and mortality can occur [3]. Moreover, due to AN’s ego-syntonic nature, individuals may feel conflicted about weight gain and recovery, which can impede and slow down the recovery process. As a result of these factors, the treatment of AN continues to pose significant challenges.

Numerous therapies have been proposed for AN treatment. Several international guidelines recommend cognitive behavioral therapy (CBT) for the psychological management of patients with eating disorders (ED) [4]. Interpersonal psychotherapy or family-based treatment, the latter especially indicated for adolescents and young adults, are two other options supported by the scientific literature [5,6]. However, a significant number of ED patients do not improve after treatment, and this is particularly relevant for those with AN. They have lower rates of recovery and experience fewer long-term effects on weight gain or psychological symptom improvement [7]. Due to the increased difficulty in recovering from AN, current treatments need to be improved. Patients with AN frequently experience anxiety and avoidance behaviors (such as food restriction) in response to certain stimuli (such as food or their own bodies). As a result, incorporating components that target the anxiety experienced by patients in relation to eating and weight gain can strengthen AN treatment. For this reason, exposure techniques have been proposed as an effective treatment for this disorder [8]. Exposure techniques are included frequently in the treatment of anxiety and related disorders, such as phobias, and are often combined with other CBT components [9]. The conceptualization of some of the core elements of AN can effectively explain the rationale of the addition of exposure therapy to the treatment of this disorder. As mentioned earlier, two of the core aspects of AN are BIDs and the fear of gaining weight (FGW). In fact, there is a possible explanation that links these two factors: the over-evaluation of weight and shape would come from the fear of imagined negative outcomes or consequences resulting from violating the thin ideal, such as being seen as disgusting, being rejected, or losing control [10]. This belief in the negative consequences of gaining weight would come with restrictive and compulsive behaviors around eating and the body that appear in EDs. In AN, restricted food intake may momentarily reduce the anxiety generated by FGW, thus reinforcing the avoidance behavior (in this case, restriction). Fear and anxiety processes are a driving force for the problematic behaviors that maintain disordered eating [10]. Another reason to pay special attention to these elements is that BIDs frequently persist even after treatment and recovery [11], and they function as a reliable predictor of relapse, which is significant in AN, as stated above [12].

Some studies have connected BIDs with the presence of attentional bias (AB), and even suggested that this connection may be bidirectional [13,14]. Body-related attentional bias is understood as the tendency to selectively attend to body appearance-related cues in preference to other information. In analyses of attentional biases related to specific parts of the body, a number of studies have found that patients with EDs, particularly those with AN and individuals with sub-clinical characteristics, pay more attention to the parts of their own body that they define as unattractive, while individuals who do not have an ED show a broader distribution of attention that is unbiased and includes the entire body [13,15,16,17,18,19,20,21,22,23]. Biased attention to body stimuli has been shown to influence the development and maintenance of ED symptoms [24], and is a mediator of the relationship between body mass index and body dissatisfaction [25]. In addition, Ferrer-Garcia et al. [26] found that body-related AB may reduce the efficacy of body exposure therapy in patients with AN. This, in turn, means that the modification of AB is a potentially important target, as it can directly affect BIDs and improve the efficacy of current treatments. 

Numerous methods have been used to assess attention bias in patients with ED. The most frequent are based on modified Stroop tasks, dot probe, visual search and eye-tracking (ET). Other methods have been used to a lesser extent, such as dichotic listening tasks, lexical decisions and spatial signaling. A thorough review of these methods and the main results achieved can be found in Jiang and Vartanian [27]. Of all these techniques, ET is the only one with which it is possible to continuously measure the attention devoted to different stimuli. On another note, and with few exceptions, most cognitive tasks involve verbal stimuli, while ET techniques facilitate the analysis of behavior that is most directly related to the stimuli that in natural situations trigger dysfunctional responses. Therefore, this technique facilitates the objective, direct measurement of attention biases. With cognitive tasks, such as those previously mentioned, these biases can only be inferred through response latencies. 

The ecological validity of attention bias assessment through ET is greater than with other methods. However, this validity can increase further if this technology is combined with another, such as virtual reality, which enables an assessment of visual attention while the participant is immersed in simulations of natural situations [28]. The first studies that used a combination of ET and VR techniques to evaluate attention biases toward diverse body parts have been published by Porras-Garcia et al. [29,30]. These studies compared the attention shown by men and women with varying levels of body dissatisfaction toward different parts of their bodies. These body areas were classified into weight-related body parts and non-weight-related body parts, based on the delimitation established in the Physical Appearance State and Trait Anxiety Scale (PASTAS) questionnaire [31]. The participants were exposed to virtual images of their own bodies, and using ET instruments incorporated into the VR devices, the number of fixations and the total time of fixation on each body part were quantified. The results showed that, regardless of the degree of body dissatisfaction, women tended to pay more attention to weight-related body parts (e.g., hips, thighs) than to non-weight-related body parts (e.g., shoulders, arms) while men were generally more attentive to non-weight-related body parts. These outcomes show that even healthy women tend to show an attention bias toward weight-related parts of the body, and suggest that this characteristic can be a risk factor that affects women to a greater degree. 

Although research on the presence of attention biases in ED is extensive, very little work has been carried out up to now to apply the information gathered in these studies to improve the efficacy of the available treatments. Among the few studies performed in this area are those of Cardi [32] and Turton [33] on reducing negative interpretation bias for social stimuli in patients with AN, and those of Boutelle [34], and Schmitz and Svaldi [35] in patients with binge-eating disorder. The modification of attention biases toward the body has not been explored in patients with AN yet. Although exposure to a mirror is frequently used in cognitive behavior therapy (CBT), its application is meant more to extinguish negative cognitive, emotional, and behavioral responses toward the patient’s own body than to directly modify attention processes [36]. Nevertheless, some results show that exposure treatments may have side effects of reducing attentional biases. In a study aimed at modifying FGW through VR exposure therapy [37], the AB in the experimental group showed a tendency to decrease.

In recent years, VR technology has progressed in its affordability and ability to create an immersive user experience. Several reviews of current literature have indicated that VR could be promising for the treatment of ED in general and AN specifically [4,10]. In explorations of VR as a potential resource to improve the treatment of ED, most studies focus on two areas: cue exposure and treatment of BIDs [38]. The incorporation of ET devices in current VR head-mounted displays (HMD) opens new possibilities related to measuring visual attention and the design of tasks that allow their modification.

The current study objective was to assess the efficacy of a task specifically aimed at reducing bodily attentional bias, based on ET and VR technology. The task included VR exposure to the person’s real body (simulated with a virtual avatar), which led the participants to gaze at different body parts for an equal amount of time. It is expected that healthy women’s AB values will become more balanced after completing the training. This implies that they will dedicate equal attention to weight-related and non-weight-related body areas. If this procedure demonstrates its effectiveness in this first study with healthy individuals, it may open up new treatment possibilities for patients with anorexia nervosa that could be explored in future studies with patients.

## 2. Materials and Methods

### 2.1. Participants

Out of the 64 initial candidates for the study, two were excluded because they reported meeting one or more exclusion criteria. In five participants, it was not possible to correctly record the eye-tracking data, and three did not complete the study as they reported feeling dizzy during the task. Finally, 54 female students from the University of Barcelona aged 20–27 participated in the study. They were recruited through campus flyers and advertisements in social network groups. Because there is a sex difference in how people perceive the size of their physical bodies, only female participants were included in the study [39]. Anorexia nervosa is a disorder whose prevalence is much higher in women than in men, and this was also a reason for including only women in our study. The exclusion criteria were (1) a self-reported diagnosis of a current ED, (2) a body mass index (BMI) of less than 17 (moderate thinness) or over 30 (obesity, according to the World Health Organization, 2004 [40]), or (3) a self-reported current severe mental disorder diagnosis (e.g., schizophrenia or bipolar disorder). (4) Visual deficits that prevent exposure, epilepsy, pregnancy and clinical cardiac arrhythmia were also considered exclusion criteria.

### 2.2. Measures

At the start of the experiment, BMI measurements were taken (weight in kilograms divided by the square of height in meters). Regarding body-related AB measures, based on the weight scale of body items from the PASTAS questionnaire and as undertaken in previous studies [25], weight-related body part areas of interest (WAOIs) were placed onto an image of a female avatar from a frontal perspective. Legs, thighs, buttocks, hips, stomach (abdomen) and waist were all included in the WAOIs. The other body parts (neck, chest, shoulders, arms and feet) were designated as non-weight-related body part areas of interest (NWAOIs). In this experiment, the participant’s head was not taken in consideration since the head of the avatar wore an HMD, similar to the participant, so the fixation of the gaze on this part of the body had more to do with the attention that this device captures than the attention actually dedicated to the head. 

The visual attention of the participants was measured by tracking the number of fixations (NF) on different parts of the body, distinguishing between WAOIs and NWAOIs, and the complete fixation time (CFT), which represents the sum of the duration of fixations. Depending on whether the participants’ visual attention was primarily directed towards weight-related areas body parts or non-weight-related body parts, CFT and NF could have either positive or negative values. Visual fixation was defined by Jacob and Karn [41] as the visual act of maintaining one’s gaze on a single location for a minimum duration, usually 100–200 ms. In this study, a duration of 100 ms was considered. These particular gaze-behavioral measurements, given by eye-tracking (ET) technology, were employed in previous studies focused on body-related attention and considered robust and continuous assessments of attention allocation toward specific body parts [21,42]. Only participants that spent more than 5 s looking at themselves in the mirror during the task were considered for the experiment.

Additionally, the participants completed the EDI-Body Dissatisfaction (EDI-BD) subscale, which measures negative attitudes towards the body and specific body areas [43]. The EDI-3 is a commonly used self-report measure for assessing ED symptoms and psychological features. The scale includes 10 items and evaluates body shape, weight, and fitness, among other factors. Another questionnaire administered was the Physical Appearance State and Trait Anxiety Scale (PASTAS) [31]. PASTAS is a reliable and valid tool for assessing both trait and state body image anxiety. Participants rated on a five-point scale ranging from 0 (never) to 5 (always) their level of anxiety about their physical appearance, including negative thoughts, tension, and physiological responses. The full-body ownership illusion (FBOI) was assessed by means of a visual analog scale (VAS). Participants were asked to rate on a scale from 0 to 100 in what extent they felt that the virtual body was their own body. Finally, fear of gaining weight (VAS-FGW) was assessed by means of a VAS from 0 (not at all) to 100 (completely). These measures were reported within the VR environment, facing a reflection of the virtual avatar.

### 2.3. Hardware and Software Features

All participants used the same VR headset to complete the task: an HTC-VIVE Pro-Eye head-mounted display (HTC Corporation, New Taipei City, Taiwan), with 2 controllers and 2 trackers. The controllers followed the participants’ hand movements, while the trackers were used to follow their feet movements. 

The VR training task and environment were developed in Unity (Unity Technologies, San Francisco, CA, USA). The environment consisted of a room with a large mirror on the wall placed 3.30 virtual meters away from the participant. The participant was situated facing her avatar. In addition, 2 boxes were placed on the floor next to the avatar’s feet, as neutral stimuli. The avatar was designed to have a simple white tank-top with jeans and black trainers (the color of the top and the jeans could be changed to match the participant’s clothing), while the hair was covered by a hat and could not be personalized. 

### 2.4. Procedure

This study was approved by the ethics committee of the University of Barcelona. Prior to the study, participants provided their informed consent by signing a form that included information on data confidentiality and the option to withdraw from the study at any point without consequences. Confidentiality was further maintained by assigning a unique identification code to each participant. Participants were told that the purpose of the study was to explore body image using virtual reality. After signing the informed consent form, participants were measured and weighed, and then completed the questionnaires.

The procedure was the same for all participants. To start, the virtual avatar was created by capturing frontal and lateral photos of the participant’s entire body. When the participant’s photo was taken and processed in our system, the experimenters manually overlapped the photograph and the virtual body by adjusting the virtual avatar’s height and various body measurements (e.g., arms, legs, hip, waist, chest, shoulder, etc.) to the participant’s silhouette. 

To evoke the full-body ownership illusion (FBOI), participants were asked to perform some movements while they looked at the avatar in the mirror in the virtual room and themselves in a first-person perspective. A visuo-tactile stimulation was carried out (see Porras-Garcia et al. [25] for a description of the full procedure). Once both procedures were finished, VAS-FGW and VAS-FBOI were assessed. 

**Figure 1:**
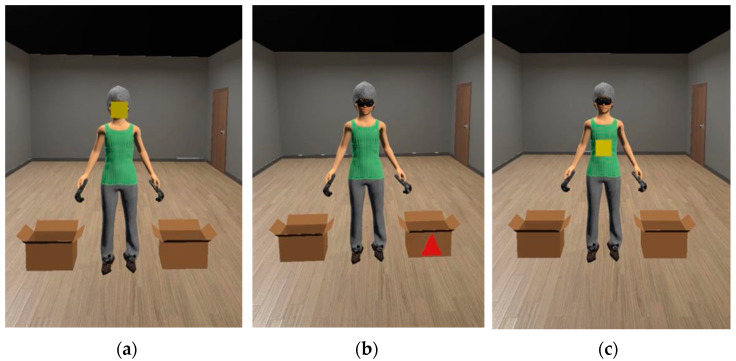
Image of the avatar placed in the scenario: (**a**) figure appearing on a non-weight-related body part (head), (**b**) figure appearing on a neutral stimulus (box on the floor), (**c**) figure appearing on weight-related body part (stomach).

Next, the first ET measurement was conducted. This consisted of staring at themselves in the mirror without moving for 30 s. This activity was masked as a sensor calibration. The masking was needed to avoid any bias due to knowledge of the true objective. It was only revealed after the procedure was completed. In order to include the data in the study, a minimum of 5 s of looking at one’s own body in the mirror was required during the task. If a participant did not look at their own body at any time during the ET recording, it was assumed that no body AB could be studied, since the body parts were not observed. After the measurement, the attentional bias modification task started. This was a VR adaptation of the procedure used by Smeets et al. [44], and it consisted of staring for 4 s at the locations at which geometrical figures appeared on the avatar’s mirror reflection, with the following distribution: 45 percent of the figures appeared on non-weight-related body parts, 45 percent on weight-related body parts, and the remaining 10% appeared on neutral stimuli (boxes on the floor) (Figure 1). The participants repeated this search-and-stare for 90 trials. Afterwards, the post-ET measurement was taken again, with the same explanation as before. The VR headset and sensors were then taken away, and the researchers dispelled any remaining doubts.

### 2.5. Data Analysis

Open Gaze and Mouse Analyzer (OGAMA) software was used to prepare the compiled ET data for the analysis. All the following analyses were carried out with SPSS (version 26). By measuring the difference between weight-related and non-weight-related AOIs, additional data transformation was performed. Accordingly, positive NF or CFT scores would indicate that the individual had spent more time gazing at the weight-related body parts than at the non-weight-related body parts, whereas negative scores would indicate the opposite.

The participants were divided into three groups for the analysis, based on the body-related AB they presented prior to the intervention. Thus, the total sample was split in three, according to the percentile distribution of the two measures used to study AB: women over the percentile 75 were assigned to the weight-related AB group (W-AB), those under the percentile 25 to the non-weight-related AB group (NW-AB) and those with CFT and NF values between those percentiles were assigned into the no AB group (No-AB). All assumptions for the analyses were met. Several analyses of variance (ANOVAs) were conducted to determine whether there was a statistically significant interaction between the time of assessment (pre- and post-intervention) and the AB defined groups. T-tests were used when necessary to decompose significant interactions. The effect sizes were reported as partial eta-squared values for ANOVAs.

## 3. Results

All participants were women, with a mean age of 22.98 ± 1.89 years and a mean BMI of 21.49 ± 2.33. The participants’ characteristics are shown in Table 1. No differences were found in age, BMI, VAS-FBOI, VAS-FGW, PASTAS and EDI-BD during the pre-intervention time at which the three AB groups (NW-AB, No-AB and W-AB) were compared.

**Figure 2:**
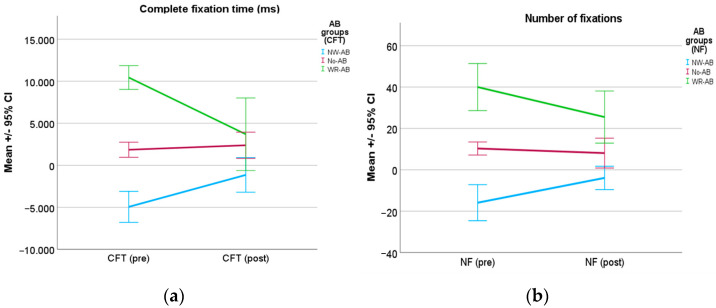
Means of the participants in the two assessment conditions (pre and post) in (**a**) complete fixation time (ms) and (**b**) number of fixations. Mean scores and 95% confidence interval are represented.

Regarding ET measures, a statistically significant interaction between the variables Time of the assessment (pre- and post-intervention) and Group (NW-AB, No-AB and W-AB) was found for CFT, F (2, 51) = 16.76, *p* < 0.001, partial η^2^ = 0.397 (Table 2). Thus, depending on the group, differences between pre–post intervention scores were found or not. As shown in Figure 2a, groups with AB (weight-related and non-weight-related) showed a tendency towards neutral values, that is, a statistically significant reduction in the AB after treatment. When the simple effects of the Group variable on the CFT measures were explored (Table 3), the pre-intervention measures showed statistically significant differences between all the groups, as expected (*p* < 0.001). However, the only statistical significance maintained in the second assessment was that between the W-AB and NW-AB groups (*p* < 0.001). Meanwhile, for the simple effects of Time, both groups W-AB and NW-AB showed a statistically significant difference between the two assessment times (*p* < 0.001; *p* = 0.006). 

When the mixed ANOVA was conducted to analyze the NF measures, a significant interaction between group and time was observed, F(2, 51) = 5.17, *p* < 0.009, partial η^2^ = 0.17 (refer to Figure 2b). The profile plot (Figure 2b) shows how the tendencies are similar to those seen with the CFT, with both AB groups getting closer to the No-AB group after the intervention. As for the simple effects of time (Table 3), the NW-AB group and the W-AB group showed a statistically significant difference (*p* = 0.048; *p* = 0.014) between the two assessment times. Regarding the simple effects of the group, the results mimic those of the CFT; while the differences were statistically significant between the groups during the first assessment (*p* < 0.001), the statistical significances maintained in the second assessment time were those between the W-AB and NW-AB groups (*p* < 0.001), and between No-AB and W-AB (*p* = 0.013).

## 4. Discussion

This study sought to assess the efficacy of a task specifically aimed at reducing body attentional bias, based on ET and VR technology. The task included VR exposure to the person’s body simulated with a virtual avatar, which led the participants to gaze at different body parts for an equal amount of time. It was expected that, after completing the training, healthy women’s AB values would become more balanced so that they would dedicate equal attention to weight-related and non-weight-related body areas. The results obtained indicate that the task was effective in reducing these biases. The women who before the treatment looked more at areas of the body related to weight and those who looked more at areas not related to weight, after performing the task looked equally at both types of body parts. However, as expected, the task did not alter the attentional pattern of women who did not display any attentional bias towards specific body parts before the treatment. The observed results follow the same line as earlier results from similar studies [44,45,46]. The AB can be modified and reduced, even if the participants are unaware of the task’s intended purpose.

As some authors have mentioned [10,24,25], having a bias towards body parts can be related to body dissatisfaction, body anxiety or even BMI. In our study, no significant differences were found between the three groups studied in the measures of body dissatisfaction (EDI-BD), body anxiety (PASTAS or FGW) or BMI. This was expected given that the sample was not clinical, although a trend can be seen in which the group with a bias towards weight-related body parts tended to score higher on body dissatisfaction than the other participants.

Some limitations should be considered. First, the nature of the AB is an issue that may benefit from further in-depth discussion and research. Although we classified participants into groups based on their most frequently gazed body parts, we did not differentiate the underlying causes of the attentional bias. The AB could have resulted from participants’ preference for certain body parts, leading them to pay more attention to those areas, while in others it could have arisen from their tendency to avoid the body parts they disliked the most. This potential differentiation was not taken into account, although it is possible that it contributed to some of the variability in the results. In another study [47], the Stroop task was used to measure attentional bias. Participants were asked to name the ink color of an ED-salient word without considering its meaning. The duration of color-naming was used as a measure of interference caused by the word meaning, which was interpreted as an indirect measure of attentional bias. However, the study found that this measure alone could not determine whether the interference was due to hypervigilance, where the participants fixated on the word longer and were distracted from the color of the word, or avoidance, where they avoided the threatening ED word and therefore the color of the word. To address this potential effect, we can consider the pre-measures of EDI-BD. Our observations revealed that participants in the W-AB group scored slightly higher on body dissatisfaction than the No-AB and NW-AB groups. Although these differences were not statistically significant, they may suggest that participants in the W-AB group have a greater inclination to glance at body parts they dislike, rather than engaging in avoidance behavior towards them.

Second, the study only involved a single session of AB modification, which may seem like a disadvantage, as repeated training sessions could potentially have a greater impact. However, recent meta-analyses by Cristea [48] and Price [49] suggest that more training sessions do not necessarily lead to better results. Nonetheless, it would be interesting to investigate the effects of multiple training sessions.

Third, although the virtual body used in the study realistically replicated the participant’s body silhouette and BMI, as well as other characteristics such as clothing color and skin tone, certain important features of overall appearance, such as hairstyle and clothing, could not be simulated. Although visuo-motor and visuo-tactile procedures were used to increase the FBOI, extending the study to additional highly realistic virtual bodies and different virtual environments could yield important insights.

After obtaining evidence of the effectiveness of the task developed to reduce attentional biases towards the body in healthy women, it is necessary to examine whether this procedure is also effective in a clinical sample. This should be the objective of a future study that, if it also demonstrates positive results, will pave the way for incorporating this procedure into the treatment of patients with eating disorders such as AN to reduce attentional biases towards the body. The reduction of biases of this type could be useful, for example, as a phase prior to exposure in treatments that use this technique to reduce anxiety towards the body and the FGW experienced by these patients. Enhancing exposure through a prior phase of reducing attentional biases towards the body could increase the effectiveness of the exposure. For this purpose, the effect of bias reduction would not need to be long-lasting, since only an immediate effect to enhance subsequent exposure would be sought. However, it would also be interesting to explore the long-term duration of the bias reduction achieved by this task, since the relationship between these attentional biases and other symptoms of anorexia nervosa could have a direct effect on them.

## 5. Conclusions

In conclusion, the attentional bias modification task using VR and ET technologies developed in this study appears to be a potential useful tool for reducing attentional bias towards different body parts in healthy women. While further research is needed to determine the effects of this technique in clinical populations, the initial findings suggest that attentional bias modification holds promise as a potential method for the treatment of anorexia nervosa. By training the participants to focus on all body parts equally, rather than only focusing on the negative or positive aspects, attentional bias modification can help shift their perspective and improve some core aspects of AN and their overall well-being. This technique could also be employed to enhance body exposure therapies for individuals with AN. The primary goal of these therapies is to eliminate negative cognitive, emotional, and behavioral responses to the body, and reducing body-related attentional biases could facilitate this process.

## Figures and Tables

**Table 1:** Descriptive measures of the three groups (non-weight-related AB, non-AB, weight-related AB).

	NW-AB Group (*n* = 13)	No-AB Group (*n* = 28)	W-AB Group (*n* = 13)
Age	22.5 ± 1.72	23 ± 1.77	23.27 ± 2.22
BMI	21.42 ± 2.15	21.86 ± 2.69	20.97 ± 1.8
EDI-BD	7.5 ±8.3	7.54 ± 6.69	10.07 ± 9.35
PASTAS	7.20 ± 7.35	5.83 ± 5.67	6.53 ± 6.50
VAS-FBOI	42 ± 27.91	59.87 ± 25.03	63.36 ± 26.68
VAS-FGW	18.13 ± 34.64	25.75 ± 27.2	25.45 ± 31.74
CFT pre	−5740.1 ± 3057.34 *	1046.32 ± 2308.99 *	9458.38 ± 2978.75 *
NF pre	−15.92 ± 14.42 *	10.3 ± 7.98 *	40 ± 19.7 *

Data are expressed as averages ± standard deviation. Abbreviations: eating disorder inventory-body dissatisfaction (EDI-BD), body anxiety (PASTAS), full-body ownership illusion (VAS-FBOI), fear of gaining weight (VAS-FWG), complete fixation time (CFT) and number of fixations (NF). Non-weight-related attentional bias (NW-AB), no attentional bias (No-AB) and weight-related attentional bias (W-AB). * *p* values < 0.05.

**Table 2:** Two-way mixed analysis of variance comparing AB groups (WR-AB, No-AB, NW-AB) with the test conditions.

	Group			Time of Assessment			Time x Group		
	F	*p*	η^2^	F	*p*	η^2^	F	*p*	η^2^
CFT	36.94	<0.001 *	0.592	1.33	0.254	0.025	16.76	<0.001 *	0.397
NF	46.94	<0.001 *	0.648	0.26	0.61	0.005	5.17	0.009 *	0.17

* *p* values < 0.05.

**Table 3:** Post hoc analyses (mean and standard deviation [SD]) on eye tracking variables for the AB groups in the two assessment times.

	NW-AB			No-AB			W-AB		
	Pre-T	Post-T		Pre-T	Post-T		Pre-T	Post-T	
	M (SD)	M (SD)	*p*	M (SD)	M (SD)	*p*	M (SD)	M (SD)	*p*
CFT	−4946.5 (6049.6)	−1148.2 (3403.4)	0.006 *	1850 (2310.8)	2381.6 (4021.8)	0.562	10,441.1 (2341)	3689.9 (7143.6)	<0.001 *
NF	−15.9 (14.4)	−3.9 (9.3)	0.048 *	10.3 (7.9)	7.1 (18.3)	0.592	40 (19.7)	25.5 (21.8)	0.014 *

Note: Complete fixation time (CFT) and number of fixations (NF). Non-weight-related attentional bias (NW-AB), no attentional bias (No-AB) and weight-related attentional bias (W-AB). * *p* values < 0.05.

## Data Availability

The datasets generated for this study are available upon request to the corresponding author.
